# A nomogram based on iron metabolism can help identify apathy in patients with Parkinson’s disease

**DOI:** 10.3389/fnagi.2022.1062964

**Published:** 2023-01-19

**Authors:** Jiang-ting Li, Yi Qu, Hong-ling Gao, Jing-yi Li, Qi-xiong Qin, Dan-lei Wang, Jing-wei Zhao, Zhi-juan Mao, Zhe Min, Yong-jie Xiong, Zheng Xue

**Affiliations:** Department of Neurology, Tongji Hospital, Tongji Medical College, Huazhong University of Science and Technology, Wuhan, China

**Keywords:** Parkinson’s disease, apathy, iron metabolism, transferrin, nomogram

## Abstract

**Backgrounds:**

Apathy is common in Parkinson’s disease (PD) but difficult to identify. Growing evidence suggests that abnormal iron metabolism is associated with apathy in PD. We aimed to investigate the clinical features and iron metabolism of apathetic patients with PD, and construct a nomogram for predicting apathy in PD.

**Methods:**

Data of 201 patients with PD were analyzed. Demographic data, Apathy Scale (AS) assessments, and serum iron metabolism parameters were obtained. Spearman correlations were used to assess relationships between AS scores and iron metabolism parameters, separately for male and female patients. Additionally, a nomograph for detecting apathetic patients with PD was built based on the results of logistic regression analysis.

**Results:**

The serum transferrin (TRF, *p* < 0.0024) concentration and total iron binding capacity (TIBC, *p* < 0.0024) were lower in the apathetic group after Bonferroni correction, and they were negatively associated with AS scores in male participants with PD (TRF, *r* = −0.27, *p* = 0.010; TIBC, *r* = −0.259, *p* = 0.014). The nomogram was developed by incorporating the following five parameters: age, sex, serum iron concentration, TIBC and Hamilton Depression Rating Scale (HAMD) scores, which showed good discrimination and calibration, with a consistency index of 0.799 (95% confidence interval = 0.732–0.865).

**Conclusion:**

Abnormal iron metabolism may contribute to apathy in PD, especially among men. TIBC levels in combination with HAMD scores can be effectively used for the prediction of apathetic patients with PD.

## 1. Introduction

Parkinson’s disease (PD) represents a progressive neurodegenerative disorder associated with a spectrum of motor and non-motor symptoms (Bloem et al., 2021). Therein, apathy is one of the most common neuropsychiatric disturbances in PD, with a prevalence of nearly 40% (den Brok et al., 2015). It can be defined as a syndrome of primary motivational loss, which cannot be attributed to emotional distress, cognitive impairment, or diminished level of consciousness (Marin, 1991). Increasing studies have shown that apathy may be a sign of PD worsening as the disease progresses (den Brok et al., 2015; Pagonabarraga et al., 2015). Apathy can be a predictor of dementia and cognitive decline over time in PD (Dujardin et al., 2009), and it is associated with more severe motor symptoms (Liu et al., 2017), lower health-related quality of life (Skorvanek et al., 2013), and heavier caregiver burden (Leroi et al., 2012; Skorvanek et al., 2013; Eglit et al., 2021).

Abnormal iron metabolism might not only contribute to the pathogenesis of PD (Dexter et al., 1989; Riederer et al., 1989; Ward et al., 2014), but also be involved in apathy (Wang et al., 2016). Neuropathological studies have shown the accumulation of iron in the substantia nigra (SN) of patients with PD, and the iron concentrations in the region increase with disease severity (Dexter et al., 1989; Riederer et al., 1989). High concentrations of iron detected in brain could induce neurotoxicity, leading to the death of dopaminergic neurons (Ward et al., 2014). Many studies have indicated that the disturbance of iron metabolism plays a crucial role in non-motor symptoms in PD, including sleep disorder (Hu et al., 2015), restless legs syndrome (Li et al., 2019), depression and anxiety (Xu et al., 2018). However, only a few studies have focused on the relationship between iron metabolism and apathy in PD. A 2016 study found that iron levels in the cerebrospinal fluid (CSF) of patients with PD were positively correlated with the Apathy Scale (AS) scores, and iron overload may be involved in apathy in PD *via* oxidative stress (Wang et al., 2016). Therefore, there is an urgent call for similar studies with larger sample sizes to explore the clinical significance of iron in apathy in PD.

Although awareness of the prevalence of apathy and its effect on clinical outcomes for patients have increased recently, the nature of apathy and the subjectivity of this neuropsychiatric symptom make the accurate diagnosis difficult (Pagonabarraga et al., 2015). Therefore, we aim to determine the features of apathy and its association with iron metabolism in the Chinese PD population, and construct a simple and useful nomogram to predict the occurrence of apathy in PD.

## 2. Materials and methods

### 2.1. Study participants

A total of 201 patients with PD participated in the study. They were inpatients or outpatients at Tongji Hospital Affiliated to Tongji Medical College of Huazhong University of Science and Technology from March 2015 to January 2022. The diagnosis of PD was defined according to the Movement Disorders Society Clinical Diagnostic Criteria (Postuma et al., 2015). Exclusion criteria were as follows: (a)patients were diagnosed with atypical Parkinsonian syndrome or secondary Parkinsonism; (b) patients had anemia, or were on treatment for anemia, such as supplemental iron, folate; (c) patients had a history of digestive-tract diseases (gastritis, gastric ulcer, hepatosis and others); and (d) patients had acute or chronic infections, tumor or serious systemic disease. Informed consent was obtained from all patients, and the study was approved by the Medical Ethics Committee of Tongji Hospital Affiliated to Tongji Medical College of Huazhong University of Science and Technology.

### 2.2. Demographic data and blood samples collection

Demographic data, including age, sex, education years, age of onset, disease duration, family history of PD and antiparkinsonian drugs, were collected, and the levodopa-equivalent daily dose (LEDD) was calculated for each participant (Tomlinson et al., 2010). Meanwhile, fasting venous blood (5 ml) was sampled and centrifuged at 4°C for 10 min (3,000 r/min). And the supernatant was separated and stored at −80°C for detection. Iron metabolism indicators, including serum iron (SI), serum ferritin (SF), transferrin (TRF), unsaturated iron-binding capacity (UIBC), total iron binding capacity (TIBC), transferrin saturation (TSAT) and soluble transferrin receptor (sTfR), were examined using the fully automated analyzer (Roche/Hitachi Cobas c 701/702, Roche Diagnostics GmbH).

### 2.3. Apathy assessments

The symptom of apathy was evaluated using the AS, which was classified as “recommended” to assess apathy in PD (Leentjens et al., 2008). The AS consists of 14 questions with four response choices to each item, corresponding to 0–3 points. The total scores range from 0 to 42 points, with higher scores indicating more severe apathy. Participants with a sum score ≥14 were classified as having a clinically meaningful apathy (Starkstein et al., 1992).

### 2.4. Motor and non-motor symptoms assessment

The clinical assessments (motor and non-motor symptoms) of each subject were completed by neurological specialists. On the one hand, the severity and stage of PD were measured using the modified Hoehn and Yahr scale (Goetz et al., 2004), and the motor function was assessed using the Movement Disorder Society-sponsored Unified Parkinson’s Disease Rating Scale (MDS-UPDRS) Part II and the MDS-UPDRS Part III (Goetz et al., 2008). On the other hand, each patient underwent a comprehensive non-motor symptoms examination: China-Modified Mini-Mental State Examination (CM-MMSE; Xu et al., 2003), Parkinson’s Disease Sleep Scale (PDSS; Chaudhuri et al., 2002). Hamilton Depression Rating Scale (HAMD; Hamilton, 1960), Hamilton Anxiety Rating Scale (HAMA; Hamilton, 1959), Scale for Outcomes in PD for Autonomic Symptoms (SCOPA-AUT; Visser et al., 2004) and Non-Motor Symptoms Scale (NMSS; Chaudhuri et al., 2007).

### 2.5. Statistical analysis

Continuous variables were described as means and standard deviations (SD), and categorical variables were described as counts and percentages. Data comparing two groups were performed using Student’s *t*-test, Mann–Whitney *U* test or chi-squared test. The Bonferroni method was applied for multiple testing. The univariate correlation analysis was performed using the Spearman rank correlations. In order to screen the predictive factors of apathy in PD, factors with *p* < 0.10 in the univariate analysis were enrolled in the multivariate stepwise logistic regression analysis with two exceptions. Age and sex were also included since they have been previously associated with apathy (Pedersen et al., 2009, 2010; Pagonabarraga et al., 2015), and the exit criteria was set at *p* > 0.10 for regression analysis. Finally, five selected predictors were used to construct a logistic regression model and then integrated into the nomogram for predicting the occurrence of apathy in PD. The discrimination ability of the model was evaluated by the consistency index (C-index) and the area under the curve (AUC) of a receiver operating curve (ROC), which ranges from 0.5 (discrimination no better than chance) to 1 (perfect discrimination). The calibration of the prediction model was conducted using a calibration plot, which estimates how close the nomogram predicted risk is to the actual observed risk. Both internal validation and calibration were performed by bootstrapping with 1,000 resamples.

All statistical analyses were performed with SPSS (version 26.0), R software (version 4.2.0) and GraphPad Prism (version 8.0). Statistical significance was defined as a two-tailed *p* < 0.05.

## 3. Results

### 3.1. Demographic and clinical characteristics

The demographic and clinical characteristics are summarized in Table 1. Briefly, 201 patients aged 59.27 years (SD = 10.72; 102 males) were enrolled in the study. A total of 99 patients (49.25%) reported apathy, while 102 patients (50.75%) reported non-apathy. Compared with non-apathetic patients, apathetic participants had higher scores of the MDS-UPDRS Part II (*p* = 0.045). There was no significant difference in age, sex, education years, disease onset, disease duration, family history of PD, LEDD, Hoehn-Yahr stage or MDS-UPDRS Part III scores between these two groups.

**Table 1:** Demographic and clinical characteristics of the study population.

Characteristic	Overall *N* = 201	Apathetic *N* = 99	Non-apathetic *N* = 102	*p*-Value
**Demographic characteristics**				
Age, years	59.27 (10.72)	60.41 (10.17)	58.16 (11.17)	0.149
Male, *n* (%)	102 (50.7)	49 (49.5)	53 (52.0)	0.727
Education, years	8.55(4.43)	8.05(4.93)	9.03(3.85)	0.303
Disease onset, years	54.75 (10.27)	55.46 (10.32)	54.06 (10.23)	0.333
Disease duration, years	5.19 (4.76)	4.97 (4.13)	5.40 (5.31)	0.511
Familial history of PD, *n* (%)	14 (7.0)	8 (8.1)	6 (5.9)	0.540
LEDD, mg	478.32 (212.73)	483.69 (236.83)	473.10 (187.43)	0.948
**Motor assessment**				
Hoehn-Yahr stage	2.23 (0.89)	2.21 (0.87)	2.25 (0.92)	0.731
MDS-UPDRS Part II	12.21 (7.82)	13.26 (7.77)	11.20 (7.77)	0.045^*^
MDS-UPDRS Part III	34.46 (16.32)	36.06 (16.48)	32.91 (16.10)	0.172
**Apathy assessment**				
AS	14.71 (12.33)	25.21 (8.33)	4.52 (4.57)	<0.001^***^
**Non-motor symptoms assessment**				
CM-MMSE	25.14 (4.68)	24.16 (5.55)	26.09 (3.41)	0.039^*^
PDSS	117.50 (22.62)	113.08 (21.96)	121.79 (25.53)	0.003^**^
HAMD	13.13 (8.03)	16.53 (7.47)	9.83 (7.15)	<0.001^***^
HAMA	12.15 (7.45)	14.53 (7.20)	9.84 (6.98)	<0.001^***^
SCOPA-AUT	10.85 (7.69)	12.55 (7.86)	9.21 (7.18)	0.002^**^
NMSS	47.31 (32.62)	56.35 (33.94)	38.54 (28.84)	<0.001^***^

AS, Apathy Scale; CM-MMSE, China-Modified Mini-Mental State Examination; HAMA, Hamilton Anxiety Rating Scale; HAMD, Hamilton Depression Rating Scale; LEDD, Levodopa Equivalent Daily Dose; MDS-UPDRS Part II, Movement Disorders Society-sponsored Unified Parkinson’s Disease Rating Scale Part II; MDS-UPDRS Part III, Movement Disorders Society-sponsored Unified Parkinson’s Disease Rating Scale Part III; NMSS, Non-Motor Symptoms Scales; PDSS, Parkinson’s disease sleep scale; SCOPA-AUT, Scale for Outcomes in PD for Autonomic Symptoms.

* *p* < 0.05;

** *p* < 0.01;

*** *p* < 0.001.

### 3.2. Serum iron metabolism

**Figure 1:**
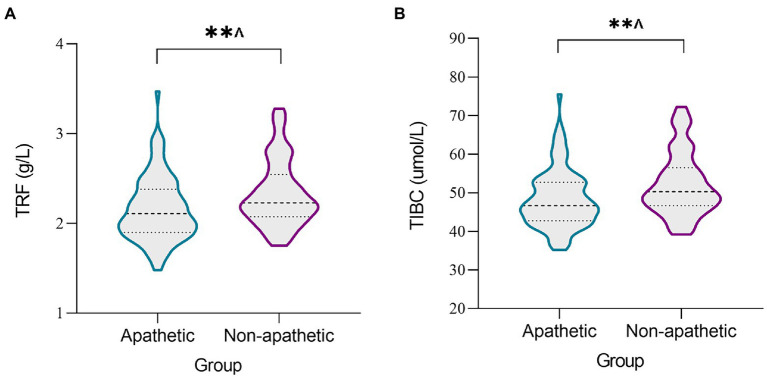
The levels of iron metabolism in serum between apathetic and non-apathetic groups. The serum concentrations of TRF **(A)** and TIBC **(B)** were lower in the apathetic group than those in the non-apathetic group. TIBC, total iron binding capacity; TRF, transferrin. **p < 0.01. ^^^The *p*-value was survived after Bonferroni correction (*p* < 0.05/21 = 0.0024).

**Figure 2:**
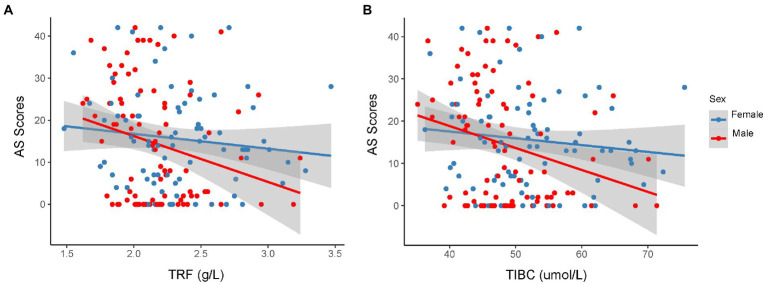
Correlation between AS scores and serum TRF **(A)** or TIBC **(B)** in the different sexual patients with PD. Spearman correlation showed a weak relationship between AS score and serum TRF concentration (*r* = −0.27, *p* = 0.010), TIBC (*r* = −0.259, *p* = 0.014) in male participants with PD. However, there were no significant associations between AS score and the level of TRF (*r* = −0.109, *p* = 0.333) or TIBC (*r* = −0.116, *p* = 0.302) for female patients. AS, Apathy Scale; PD, Parkinson’s disease; TIBC, total iron binding capacity; TRF, transferrin.

Both TRF concentration (*p* < 0.0024) and TIBC levels (*p* < 0.0024) were lower in the apathetic group than in the non-apathetic group after Bonferroni correction (Table 2; Figure 1). No significant changes were observed in the levels of SI, SF, UIBC, TSAT or sTfR between the two groups. When stratified by gender, lower TRF concentration (*r* = −0.27, *p* = 0.010) and TIBC (*r* = −0.259, *p* = 0.014) were significantly associated with lower AS scores in male participants with PD (Figure 2). However, no correlations were not found for female patients (TRF, *r* = −0.109, *p* = 0.333; TIBC, *r* = −0.116, *p* = 0.302).

**Table 2:** The levels of iron metabolism in serum between apathetic and non-apathetic groups.

Iron metabolism	Overall *N* = 171	Apathetic *N* = 87	Non-apathetic *N* = 84	*p*-Value
SI, μmol/L	16.25 (5.66)	15.43 (4.99)	17.10 (6.20)	0.054
SF, μg/L	177.56 (123.94)	183.18 (123.94)	171.74 (124.42)	0.423
TRF, g/L	2.24 (0.37)	2.16 (0.36)	2.33 (0.37)	0.002^**^^,^^^^
UIBC, μmol/L	34.94 (9.25)	32.92(7.78)	34.99 (10.50)	0.159
TIBC, μmol/L	50.23 (8.06)	48.36(7.55)	52.17 (8.15)	0.002^**^^,^^^^
TSAT, %	32.59 (11.34)	32.27 (9.83)	32.92 (12.77)	0.770
sTfR, mg/L	2.80 (0.71)	2.76 (0.61)	2.85 (0.80)	0.664

SF, serum ferritin; SI, serum iron; TRF, transferrin; sTfR, soluble transferrin receptor; TIBC, total iron binding capacity; TSAT, transferrin saturation, UIBC, unsaturated iron binding capacity.

** *p* < 0.01.

^ The *p*-value was survived after Bonferroni correction (*p* < 0.05/21 = 0.0024).

### 3.3. Apathy and other non-motor symptoms

The mean (SD) AS scores for the apathetic and the non-apathetic patients were 25.21 (8.33) and 4.52 (4.57), respectively (*p* < 0.001). Overall, Patients with apathy showed more severe non-motor symptoms, including cognitive function, sleep disturbance, depression, anxiety and autonomic dysfunction (Table 1). Apathetic patients had significantly lower scores for the CM-MMSE (*p* = 0.039) and PDSS (*p* = 0.003), and higher scores for the HAMD, HAMA, SCOPA-AUT, and NMSS (all *p* < 0.05) compared to non-apathetic patients. For cognitive domains, apathetic patients showed poorer performance on executive function (*p* = 0.009), attention and calculation abilities (*p* = 0.030) than non-apathetic patients (Supplementary Table 1).

### 3.4. Factor selection for the predictive model, validation and calibration of the nomogram

**Figure 3:**
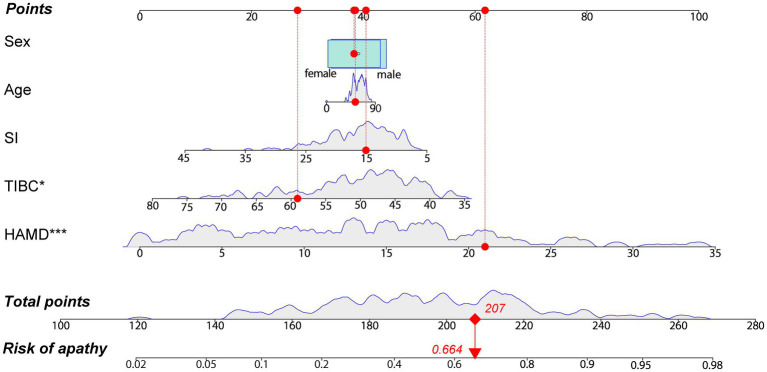
A constructed nomogram for the prediction of a patient with apathy. The nomogram was developed by incorporating the following five parameters: age (years), sex (male, female), SI concentration (μmol/L), TIBC (μmol/L) and HAMD scores. For each patient, a value of related variable is situated on the variable axis of the nomogram, and a vertical upward line determines the points calculated for each variable. Sum the points for each variable and draw a line from the total points axis to determine apathy probabilities. The red line and dots depict an example for predicting the probability of having apathy for a 53 years old female with PD, who has a SI level of 15.06 μmol/L, a TIBC level of 59.06 μmol/L and HAMD scores of 21. HAMD, Hamilton Depression Rating Scale; SI, serum iron; TIBC, total iron binding capacity. *p < 0.05, ***p < 0.001.

The correlation between other variables and AS scores are shown in Supplementary Table 2. The stepwise logistic regression analysis indicated that the concentration of SI (*OR* = 0.943, 95% CI = 0.884–1.007, *p* = 0.079), TIBC (*OR* = 0.935, *95% CI* = 0.891–0.981, *p* = 0.006) and HAMD (*OR* = 1.165, 95% CI = 1.102–1.232, *p* < 0.001) scores can be identified as the risk factors for apathy in PD (Supplementary Table 3). After taking the influence of age and sex into account, the final logistic model incorporated five predictors (age, sex, SI concentration, TIBC, and HAMD scores) and was developed as a nomogram that is represented in Figure 3. For each patient, higher total points indicated a higher risk of apathy. The red line and red dots on the graph show an example of using the nomograph to predict the probability of apathy for a given patient with PD.

**Figure 4:**
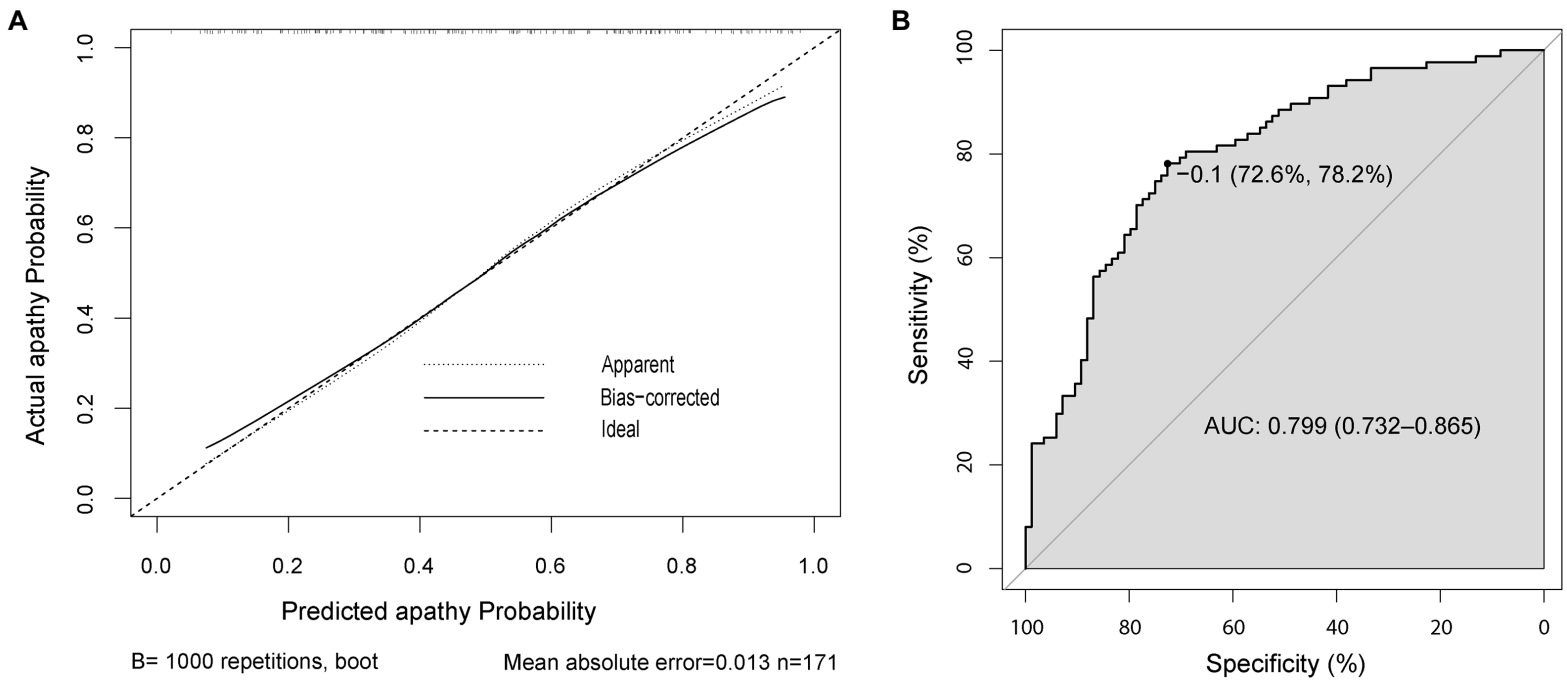
The calibration and accuracy of the nomogram. Calibration curve for predicting the probability of apathetic patients with PD **(A)**. ROC curve of the predictive model showing an AUC of 0.799 (95% CI = 0.732–0.865) **(B)**. AUC, area under the curve; CI, confidence interval; PD, Parkinson’s disease; ROC, receiver operating characteristic.

Furthermore, the internal validation was performed by using the bootstrap method with 1,000 resamples to validate the performance of the nomogram. And the calibration curve (Figure 4A) indicated a preferable consistency between the predicted model and the ideal model. The C-index was 0.799 (95% CI = 0.732–0.865), and the correspondent ROC curve and its AUC were provided in Figure 4B, reflecting a good discrimination ability of the nomogram.

## 4. Discussion

Apathy was common in our Chinese cohort, which is associated with more severe motor and non-motor symptoms. To the best of our knowledge, lower serum TRF levels and TIBC were observed in apathetic patients with PD for the first time, and both were negatively associated with AS scores in male patients with PD. Therefore, the disturbance of iron metabolism may contribute to apathy in PD, especially among male patients. More importantly, we developed an objective and practical nomogram to assist clinicians in screening apathetic patients with PD.

About 49.25% of the 201 patients were apathetic in our study, which is similar to the findings of a Chinese study on 145 patients assessed by AS (Wang et al., 2016). The subjective motor experiences of daily living in apathetic patients were impaired, implying that these patients exhibit a more severe neurodegenerative pattern progression (den Brok et al., 2015; Pagonabarraga et al., 2015). Also, dopamine ascending pathways were reported to play critical roles in the pathophysiology of apathy in PD (Levy and Czernecki, 2006).

We also found that apathy in PD was related to worse cognitive function, which is consistent with previous studies (Liu et al., 2017; Brown et al., 2019). Neuroimaging studies have demonstrated that cognitive decline in apathetic patients with PD is strongly linked with significant gray and white matter loss in the frontal lobe areas and the insular region (Alzahrani et al., 2016). In addition, apathetic patients with PD had more severe depression and anxiety, worse sleep quality and more severe autonomic dysfunction than non-apathetic patients. These aspects may probably account for the poorer quality of life experienced by apathetic compared to non-apathetic patients, as previously reported (Skorvanek et al., 2013).

As far as we known, the difference in serum TRF concentration or TIBC between PD participants with and without apathy has not been reported yet. TRF is a glycoprotein that circulates iron in a soluble, non-toxic form and delivers it to other body tissues (Gkouvatsos et al., 2012), including the brain (Moos et al., 2007). Another biomarker, TIBC, is regarded as a parameter to evaluate the maximal capacity of serum to transport iron, which is proportional to the concentration of TRF in serum (Kasvosve and Delanghe, 2002). Due to the decreased TRF concentration, a lack of iron load to transferrin may allow non-transferrin-bound iron enters the pool of labile iron (Ashraf et al., 2020), leading to enhanced harmful lipid peroxide production. It was considered one of the reasons contributing to the death of dopaminergic neurons in PD (Ma et al., 2021). Furthermore, similar research has also revealed that iron level in the CSF is significantly higher and positively correlated with·OH level in apathetic patients with PD. The authors also speculated excessive iron may be involved in apathy *via* oxidative stress (Wang et al., 2016). However, the association between central and peripheral iron in PD has not been fully elucidated (Costa-Mallen et al., 2017; Ashraf et al., 2020). Costa-Mallen et al. (2017) found decreased iron in circulation in favor of iron accumulation in the substantia nigra pars compacta (SNpc). In this investigation, levels of TRF and TIBC were significantly lower in apathetic patients than that in non-apathetic patients, based on which, we speculated that the decrease in serum TRF level in apathetic patients may result from the accumulation of iron in the brain, suggesting that disrupted iron metabolism may be involved in apathy in PD.

In male patients with PD, further analyses indicated that AS scores increased with decreased TRF concentration or TIBC. Recently, there has been increased interest in sex-related differences in PD or brain iron metabolism. An imaging study conducted on healthy individuals showed a selective lower total subcortical brain iron levels in middle-aged women compared to men and young women (Persson et al., 2015). Previous studies have also demonstrated the regulatory role of estrogen in brain iron metabolism (Sohrabji, 2007; Grubić Kezele and Ćurko-Cofek, 2020). Tzu-Yun et al. found that estradiol *via* different estrogen receptors could diminish the iron overload-induced autophagy and injury in female and male mice, respectively (Chen et al., 2019). Furthermore, the results by Wang and colleagues suggested that the striatum of male mice was more sensitive to iron accumulation than that of female mice. This trend was similar to those previously observed in clinical studies, which showed that men have a much higher risk of developing PD than women (Grubić Kezele and Ćurko-Cofek, 2020), and they are more likely to be apathetic (Pagonabarraga et al., 2015). Sex-associated differences are common among patients with PD, and we believe that this would be valuable for a future study.

The other particular strength of our study is that we developed a novel practical model to identify patients with PD who had a high risk of apathy. Based on standard deviation along the nomogram, both TIBC concentration and HAMD scores were important predictors, while age, sex and SI concentration do not appear to play a role in predicting apathy in this model. Our findings revealed that patients with a lower TIBC level were at a higher risk of apathy. Meanwhile, a trend towards decreased serum iron was found in apathetic patients although this difference did not reach statistical significance. Lower serum iron may also be linked with apathy. Patients with iron deficiency anemia could present with apathy (Bourre, 2006). And a study on psychiatric patients also found that oral iron treatment could bring about a reduction in apathy and the likelihood of resorting to psychiatric admission (Kassir, 2017). TRF delivers iron to the brain from the blood by binding to TRF receptors at the blood–brain barrier (Ma et al., 2021). As discussed previously, the relationship between peripheral and central iron in PD is still unclear and some points of view are contradictory. A previous study showed that lower serum iron in patients with PD may result from progressive iron deposition in the SNpc (Costa-Mallen et al., 2017). Therefore, we speculated that lower TIBC and the trend towards decreased serum iron in apathetic patients might provide indirect evidence for brain iron overload. And the specific mechanism may involve the regulation of TRF receptors expression on the cells of the blood–brain barrier (Ma et al., 2021). However, both iron deficiency in peripheral and central systems were simultaneously observed in patients with PD by Piao et al. (2017). In summary, the disturbance of iron metabolism is definitively involved in the pathogenesis of PD, and may also play a role in apathy although further investigation is required.

In addition to TIBC level, patients with severe depression were also more likely to be apathetic, which is consistent with the study from Brown et al. (2019). A 4-year prospective cohort study demonstrated that a high Hamilton Depression Rating Scale (HDRS) score was a predictor of apathy in PD (Ou et al., 2020), which may result from shared pathophysiological mechanisms. Maillet et al. found that the serotonergic alteration within the bilateral ventral striatum and the right caudate nucleus may play a role in apathy as well as depression (Maillet et al., 2016). Moreover, it is worth noting that we did not confuse apathy with depression, although there is a large overlap between apathy and depression (Pagonabarraga et al., 2015). The results from the nomogram should be viewed with caution, that means when patients with PD have both depression and iron metabolism abnormality, clinicians need to be alert to the possibility of apathy.

Since apathy is frequently associated with greater cognitive decline, more severe motor symptoms and heavier caregiver burden (Pagonabarraga et al., 2015), it is a need for earlier intervention. But there is no standard treatment for apathy. Acetylcholinesterase inhibitors and dopamine agonists were reported to improve apathy in PD (Seppi et al., 2019). Based on our study, we believed that maintaining iron homeostasis could be a potential therapeutic target for apathy in PD. Notably, the results should be interpreted with caution and there is much work needed to be carried out.

Several limitations of our study should be acknowledged. Firstly, this is a single-center study evaluating Chinese patients, which needs multicenter clinical datasets to perform the external validation of the nomogram. Secondly, further longitudinal studies are warranted to determine predictors of apathy. Thirdly, cognitive function of each participant was evaluated only by CM-MMSE, and more cognitive tests such as the Montreal Cognitive Assessment should be used in future studies. Finally, the analysis presented here has not incorporated radiological findings of patients, which will help to further explore the neural structures and levels of iron in the brain related to apathetic patients with PD.

## 5. Conclusion

In summary, apathy in PD is related to more severe motor and non-motor impairment. Lower serum TRF concentration and TIBC were first reported in apathetic patients with PD, and both were negatively associated with AS scores in male patients with PD. These findings suggest that abnormal iron metabolism may serve as an underlying mechanism of apathy in PD. TIBC level in combination with HAMD scores could be effectively used for the prediction of apathy in PD.

## Data availability statement

The original contributions presented in the study are included in the article/Supplementary material, further inquiries can be directed to the corresponding authors.

## Ethics statement

The studies involving human participants were reviewed and approved by the Medical Ethics Committee of Tongji Hospital Affiliated to Tongji Medical College of Huazhong University of Science and Technology. The patients/participants provided their written informed consent to participate in this study.

## Author contributions

ZX, Y-jX, and J-tL contributed to study design. J-tL, YQ, H-lG, J-yL, Q-xQ, D-lW, and J-wZ contributed to data acquisition. J-tL, Z-jM, ZM, Y-jX, and ZX analyzed and extracted the data. J-tL, YQ, H-lG, Y-jX, and ZX wrote the manuscript. All authors contributed to the article and approved the submitted version.

## Funding

This work was supported by the National Natural Science Foundation of China (grant number 81771376).

## Conflict of interest

The authors declare that the research was conducted in the absence of any commercial or financial relationships that could be construed as a potential conflict of interest.

## Publisher’s note

All claims expressed in this article are solely those of the authors and do not necessarily represent those of their affiliated organizations, or those of the publisher, the editors and the reviewers. Any product that may be evaluated in this article, or claim that may be made by its manufacturer, is not guaranteed or endorsed by the publisher.
