# Supplementary material for: A nomogram based on iron metabolism can help identify apathy in patients with Parkinson’s disease
Source: Front Aging Neurosci. 2023 Jan 19;14:1062964. doi: 10.3389/fnagi.2022.1062964 (PMC9892642; doi:10.3389/fnagi.2022.1062964)
Supplement: Supplementary file 1 [file Table_1.docx]

Supplementary Material

**Supplementary Table 1. The cognitive function assessments in patients with PD**

| Domain | Overall  N = 201 | Apathetic  N = 99 | Non-apathetic  N = 102 | *p*-value |
| --- | --- | --- | --- | --- |
| CM-MMSE |  |  |  |  |
| Total score | 25.14 (4.70) | 24.16 (5.55) | 26.09 (3.41) | 0.039^*^ |
| Executive | 8.98 (1.59) | 8.56 (1.98) | 9.38 (0.94) | 0.009^**^ |
| Registration | 2.86 (0.46) | 2.82 (0.54) | 2.89 (0.37) | 0.254 |
| Attention and calculation | 2.94 (2.03) | 2.58 (2.16) | 3.28 (1.84) | 0.030^*^ |
| Recall | 2.21 (0.95) | 2.22 (0.95) | 2.20 (0.94) | 0.795 |
| Language and praxis | 7.19 (1.72) | 6.78 (1.95) | 7.59 (1.35) | 0.005 |

*Abbreviations:* *CM-MMSE*, China-Modified Mini-Mental State Examination; *PD*, Parkinson’s Disease. ^*^ *p*<0.05, ^**^ *p*<0.01.
