# Supplementary material for: A nomogram based on iron metabolism can help identify apathy in patients with Parkinson’s disease
Source: Front Aging Neurosci. 2023 Jan 19;14:1062964. doi: 10.3389/fnagi.2022.1062964 (PMC9892642; doi:10.3389/fnagi.2022.1062964)
Supplement: Supplementary file 2 [file Table_2.docx]

Supplementary Material

**Supplementary Table 2. Univariate analyses of variables associated with apathy in PD**

| Characteristic | *r* | *p*-value |
| --- | --- | --- |
| Demographic characteristics |  |  |
| Age, years | 0.102 | 0.150 |
| Sex, n (%) | 0.025 | 0.728 |
| Education, years | -0.073 | 0.304 |
| Disease onset, years | 0.059 | 0.402 |
| Disease duration, years | -0.046 | 0.512 |
| Familial history of PD, n (%) | 0.043 | 0.543 |
| LEDD, mg | -0.005 | 0.948 |
| Motor assessment |  |  |
| Hoehn-Yahr stage | -0.024 | 0.732 |
| MDS-UPDRS Part II | 0.142 | 0.045* |
| MDS-UPDRS Part III | 0.093 | 0.190 |
| Non-motor symptoms assessment |  |  |
| CM-MMSE | -0.146 | 0.039* |
| PDSS | -2.12 | 0.002** |
| HAMD | 0.425 | ＜0.001*** |
| HAMA | 0.331 | ＜0.001*** |
| SCOPA-AUT | 0.219 | 0.002** |
| NMSS | 0.281 | ＜0.001*** |
| Iron metabolism |  |  |
| SI, umol/L | -0.134 | 0.080 |
| SF, ug/L | 0.061 | 0.424 |
| TRF, g/L | -0.239 | 0.002** |
| UIBC, umol/L | -0.108 | 0.159 |
| TIBC, umol/L | -0.243 | 0.001** |
| TSAT, % | -0.022 | 0.771 |
| sTFR, mg/L | -0.033 | 0.665 |

*Abbreviations: AS*, Apathy Scale; *CM-MMSE*, China-Modified Mini-Mental State Examination; *ESS*, Epworth Sleepiness Score; *HAMA*, Hamilton Anxiety Rating Scale; *HAMD*, Hamilton Depression Rating Scale; *LEDD*, Levodopa Equivalent Daily Dose; *MDS-UPDRS Part II*, Movement Disorders Society-sponsored Unified Parkinson’s Disease Rating Scale Part II; *MDS-UPDRS Part III*, Movement Disorders Society-sponsored Unified Parkinson’s Disease Rating Scale Part III; *NMSS*, Non-Motor Symptoms Scales; *PD*, Parkinson’s Disease; *PDSS*, Parkinson’s Disease Sleeping Scale; *SCOPA-AUT*, Scale for Outcomes in PD for Autonomic Sympto*ms; SF*, serum ferritin; *SI*, serum iron; *TRF*, transferrin; *sTfR*, soluble transferrin receptor; *TIBC, total iron binding capacity*; *TSAT, transferrin saturation*, *UIBC*, *unsaturated iron binding capacity.* ^*^ *p*<0.05, ^**^ *p*<0.01, ^***^ *p*<0.001.
