# Supplementary material for: A nomogram based on iron metabolism can help identify apathy in patients with Parkinson’s disease
Source: Front Aging Neurosci. 2023 Jan 19;14:1062964. doi: 10.3389/fnagi.2022.1062964 (PMC9892642; doi:10.3389/fnagi.2022.1062964)
Supplement: Supplementary file 3 [file Table_3.docx]

Supplementary Material

**Supplementary Table 3. The multivariate logistic regression analysis for exploring factors of apathetic patients with PD**

| Predictors | *B* | *SE* | *OR* | 95%*CI* | *p-value* |
| --- | --- | --- | --- | --- | --- |
| SI, umol/L | -0.058 | 0.033 | 0.943 | 0.884-1.007 | 0.079 |
| TIBC, umol/L | -0.067 | 0.025 | 0.935 | 0.891-0.981 | 0.006** |
| HAMD | 0.153 | 0.029 | 1.165 | 1.102-1.232 | < 0.001*** |

*Abbreviations: HAMD*, Hamilton Depression Rating Scale; *PD*, Parkinson’s Disease; *SI*, serum iron; *TIBC, total iron binding capacity.* ^**^ *p*<0.01, ^***^ *p*<0.001.
